# Inhibition of Aquaporin-4 Improves the Outcome of Ischaemic Stroke and Modulates Brain Paravascular Drainage Pathways

**DOI:** 10.3390/ijms19010046

**Published:** 2017-12-23

**Authors:** Ionica Pirici, Tudor Adrian Balsanu, Catalin Bogdan, Claudiu Margaritescu, Tamir Divan, Vacaras Vitalie, Laurentiu Mogoanta, Daniel Pirici, Roxana Octavia Carare, Dafin Fior Muresanu

**Affiliations:** 1 Department of Human Anatomy, University of Medicine and Pharmacy of Craiova, Craiova 200349, Romania; danapirici@yahoo.com; 2 Department of Physiology, University of Medicine and Pharmacy of Craiova, Craiova 200349, Romania; adibalseanu@yahoo.com (T.A.B.); bogdan.catalin@yahoo.co.uk (C.B.); 3 Department of Pathology, University of Medicine and Pharmacy of Craiova, Craiova 200349, Romania; c_margaritescu2000@yahoo.com; 4 Department of Research Methodology, University of Medicine and Pharmacy of Craiova, Petru Rares Street 2, Craiova 200349, Romania; divantamir@gmail.com; 5 Department of Clinical Neurosciences, University of Medicine and Pharmacy “Iuliu Hatieganu”, Cluj-Napoca 400000, Romania; vvacaras@umfcluj.ro (V.V.); dafinm@ssnn.ro (D.F.M.); 6 Department of Histology, University of Medicine and Pharmacy of Craiova, Craiova 200349, Romania; laurentiu_mogoanta@yahoo.com; 7 Faculty of Medicine, University of Southampton, Southampton SO17 1BJ, UK; r.o.carare@soton.ac.uk

**Keywords:** aquaporin-4 inhibition, ischemic stroke, non-reperfusion ischemia, basement membranes, paravascular drainage

## Abstract

Aquaporin-4 (AQP4) is the most abundant water channel in the brain, and its inhibition before inducing focal ischemia, using the AQP4 inhibitor TGN-020, has been showed to reduce oedema in imaging studies. Here, we aimed to evaluate, for the first time, the histopathological effects of a single dose of TGN-020 administered after the occlusion of the medial cerebral artery (MCAO). On a rat model of non-reperfusion ischemia, we have assessed vascular densities, albumin extravasation, gliosis, and apoptosis at 3 and 7 days after MCAO. TGN-020 significantly reduced oedema, glial scar, albumin effusion, and apoptosis, at both 3 and 7 days after MCAO. The area of GFAP-positive gliotic rim decreased, and 3D fractal analysis of astrocytic processes revealed a less complex architecture, possibly indicating water accumulating in the cytoplasm. Evaluation of the blood vessels revealed thicker basement membranes colocalizing with exudated albumin in the treated animals, suggesting that inhibition of AQP4 blocks fluid flow towards the parenchyma in the paravascular drainage pathways of the interstitial fluid. These findings suggest that a single dose of an AQP4 inhibitor can reduce brain oedema, even if administered after the onset of ischemia, and AQP4 agonists/antagonists might be effective modulators of the paravascular drainage flow.

## 1. Introduction

Stroke has become the second leading cause of death above the age of 60 years worldwide, and the fifth leading cause of death in the age range of 15–59 years [1,2]. Ischemic stroke results from the lack of blood flow to the brain, and accounts for up to 80% of all stroke patients [1,2]. To date, the only Food and Drug Administration (FDA)-approved physiopathological therapy for ischemic stroke remains the use of tissue plasminogen activator (tPA), and mechanical clot retrieval to recanalize occluded blood vessels. Hypoxia that appears after a stroke leads to multiple profound changes in the blood-brain barrier (BBB), by destabilization of the intercellular endothelial adhesion molecules, displacement of the astrocytic end-feet, and the inflammatory cascade that follows [3]. Specifically for oedema, the most important acute complication of increased BBB permeability following a hypoxic-ischemic event, for which there is no targeted treatment yet. The only currently accepted non-surgical approach in the treatment of brain oedema is parenteral administration of hypertonic solutions, which lower intracranial pressure, by shifting water from the brain to the blood [4]. However, even this treatment may be complicated by hypovolemia, electrolyte disturbances, and nephrotoxicity with prolonged use [5].

Aquaporin-4 (AQP4), a member of the water channel family, is most abundant in the brain, acting as a passive osmotic-driven bidirectional water gateway, and plays essential roles in mediating water equilibrium and the pathogenesis of brain oedema [6,7,8,9]. AQP4-formed channels are concentrated especially in the astrocytes, from the perivascular end-feet to the whole astrocyte membrane, as well as in the periventricular and subpial glia limitans [10,11,12]. In experimental mouse models of vasogenic brain oedema, such as intraparenchymal fluid infusion, cortical freeze injury, obstructive hydrocephalus, and brain abscess, AQP4 facilitates brain water efflux [10,11]. AQP4 knockout mice under these conditions show increased tissue oedema, higher intracranial pressure, and worse clinical outcomes compared to wild type animals [13,14,15]. In contrast, induced cytotoxic oedema (cellular swelling with an intact blood-brain barrier), as in focal cerebral ischemia without hemorrhage, leads to improved clinical outcomes and reduced brain swelling in AQP4 knockout mice compared to wild type animals [16]. In the same conditions, however, AQP4-overexpressing mice showed accelerated cytotoxic brain swelling compared to wild type animals [17].

In vitro and in vivo studies identified 2-(nicotinamide)-1,3,4-thiadiazole (TGN-020) as a potent AQP4 inhibitor [18,19,20]. Although pre-treatment with a single dose of TGN-020 before the ischemic event showed no toxicity or associated histopathological changes while reducing brain oedema and infarct volumes in a mouse model of cerebral ischemia, no study has yet characterized the mechanisms that drive these effects [19,20].

In the present study, we tested the hypothesis that TGN-020 administered within 15 min of middle cerebral artery occlusion (MCAO), results in reduced oedema, albumin extravasation, gliosis and apoptosis, at 3 and 7 days after insult. We assessed possible mechanisms by which TGN-020 may prevent solutes from entering the brain parenchyma, mechanisms that, if controlled, might present new treatment options for stroke and some neurodegenerative diseases.

## 2. Results

### 2.1. Motor Testing for Treated/Untreated Animals after MCAO

**Figure 1:**
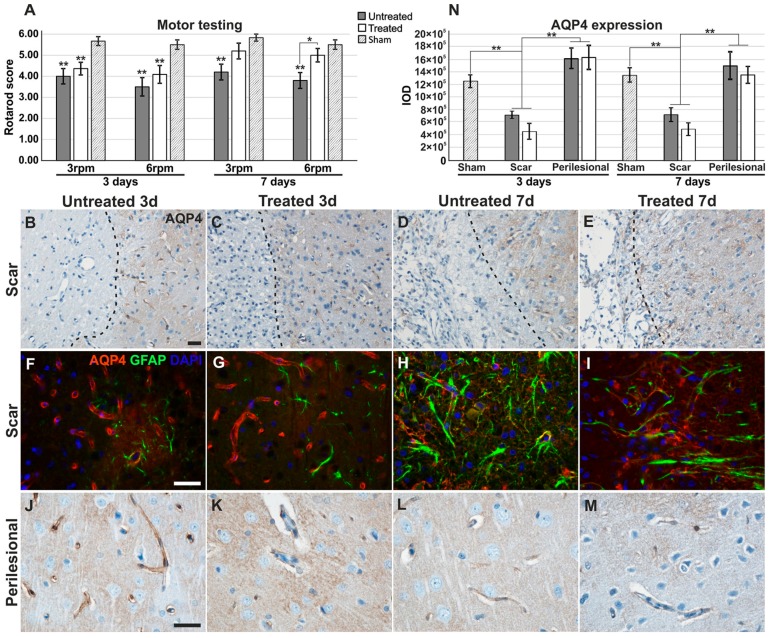
Motor testing and comparative aquaporin expression patterns. (**A**) Motor performances showed an overall increase for TGN-020-treated animals, with a significant difference for the 6 rpm testing at 7 days; (**B**–**I**) Aquaporin-4 (Aqp4) expression is decreased in the glial feltwork around the infarct tissue while still retaining strong vascular expression, and in perilesional regions (**J**–**M**) it is more intense and diffusely present in the glial feltwork, both for 3-day treated and untreated animals. At 7 days, Aqp4 is present around the infarct, less in the blood vessels and more in the astrocytes forming the more robust scar, and is overall, more intense in untreated animals (**D**,**E**,**H**,**I**); (**J**–**M**) Further from the infarct, it is diffuse in the neuropil for the untreated animals and more petechial in the treated rats; (**N**) Integrated optical density (IOD) of the immunohistochemistry staining shows significant lower values for scar regions for both treated and untreated animals at both time points. Data are expressed as the means ± SEM, * *p* < 0.05, ** *p* < 0.01 using a one-way ANOVA followed by a post hoc Fisher’s LSD test, *n* = 5–6/group. Unless indicated, significance is shown for differences from the measurements in sham animals. Dotted lines in the micrographs delineate the infarct areas. Scale bars represent 20 µm.

There was a tendency for all TGN-020-treated animals to exhibit higher motor scores compared to non-treated counterparts (Figure 1A). At 3 days, both treated and untreated rats showed significantly lower motor scores than the sham animals, while at 7 days, the difference for the treated animals was not deemed significant. At 7 days, rotarod scores were significantly higher for TGN-020-treated animals (5.00 ± 0.71) than for untreated animals (3.80 ± 0.84) at 6 rpm (*p* < 0.05).

### 2.2. Aquaporin-4 Expression in the Stroke Model

After confirming the histopathology of ischemic stroke with minimal hemorrhagic transformations (see Supplementary Results, Figure S1), we have first sought to evaluate the expression of AQP4 in these animals. In the brains of 3-day untreated animals, the reactivity was intense in the blood vessel walls in the forming glial scar, but less abundant between them, while in the surrounding ipsilateral neuropil, it became more diffuse and less centered on blood vessels, compared to sham animals (see above; Figure 1B,F,J). The same pattern was also found in TGN-020 treated animals (Figure 1C,G,K).

In the glial scars of 7-day untreated animals (Figure 1D,H), the reactivity decreased in the blood vessel walls, but it was expressed higher around them in the reactive astrocytes present at this time (Figure 1H). In the distant perilesional areas, the reactivity was also relatively low in the blood vessels, but it was more diffuse in the neuropil compared to the previous time point (Figure 1L). In the glial scars of TGN-020-treated animals, the reactivity was also reduced in the blood vessels and increased in the glial feltwork (Figure 1E,I). However, in the perilesional areas, AQP4 expression was less diffuse in the neuropil, more petechial and sometimes seemingly aggregated more around blood vessels (Figure 1M). Evaluation of the overall AQP4 staining intensity/area as the integrated optical density (IOD; see Supplementary Materials and Methods) showed that indeed, in the scar region, there is a significant decrease of expression for both treated and untreated animals (Figure 1N). Although the difference was not statistically significant, treated animals had a tendency for lower IOD values compared to the untreated group.

### 2.3. Reduced Albumin Extravasation in TGN-020-Treated Animals

**Figure 2:**
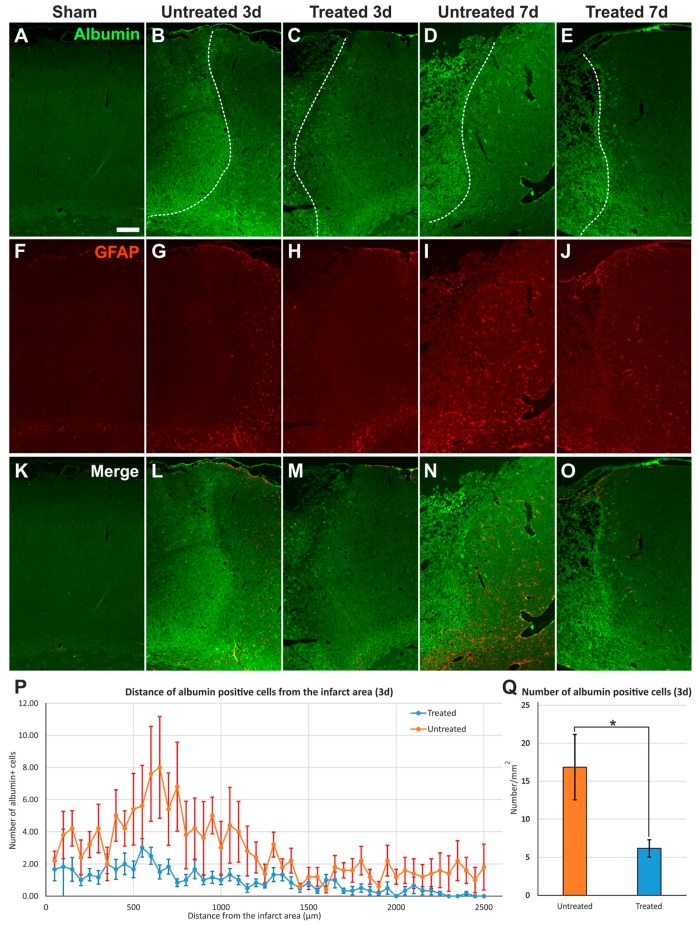
Albumin infiltration in perilesional cortex. (**A**–**O**) Immunostaining for endogenous rat albumin reveals infiltration in infarct areas and cellular silhouettes in the surrounding parenchyma, apparently more in untreated animals than in TGN-020-treated rats, and overall, reduced gliosis in treated animals compared to untreated animals; (**P**) Distance frequency distribution of albumin-positive cellular silhouettes shows more frequent elements for the untreated animals around the infarct, with a peak at 500–1000 µm from the necrotic tissue (Student’s *t*-test, *p* < 0.001), at 3 days; (**Q**) The total number of albumin-positive cells quantified in (**P**) shows a significantly higher density for untreated rats (Student’s *t*-test, *p* < 0.05). Data are expressed as the means ± SEM. The dotted lines delineate the infarct cores on the left side in images **B**–**E**. Scale bars in the micrographs represent 200 µm. * *p* < 0.05

Next, we analyzed scanned images of whole hemispheres captured for GFAP and extravasated endogenous rat albumin (Figure 2).

While there was less albumin signal in sham/control animals, there was abundant albumin infiltration in the infarct core, scar, and surrounding tissues, with an apparent tendency of TGN-020-treated animals to exhibit less albumin diffusion and bordering astrogliosis, compared to untreated MCAO animals for both 3- and 7-day follow-up times (Figure 2). The frequency distribution of albumin-positive cells around the infarct core showed more silhouettes in untreated animals than in treated animals, with a peak difference for the 500–1000 µm distance interval (Figure 2P). Even considering the overall counts, the difference between the two animal groups was statistically significant for the complete studied interval (*p* < 0.001). Regarding the average total number of albumin-stained cellular silhouettes normalized to 1 mm^2^, the untreated animals (16.85 ± 4.30/mm^2^) also showed a significant higher density compared to TGN-020-injected animals (6.16 ± 1.11/mm^2^) (*p* < 0.05) (Figure 2Q).

### 2.4. Reduced Gliosis and Different Glial Morphology in TGN-020-Treated Animals

**Figure 3:**
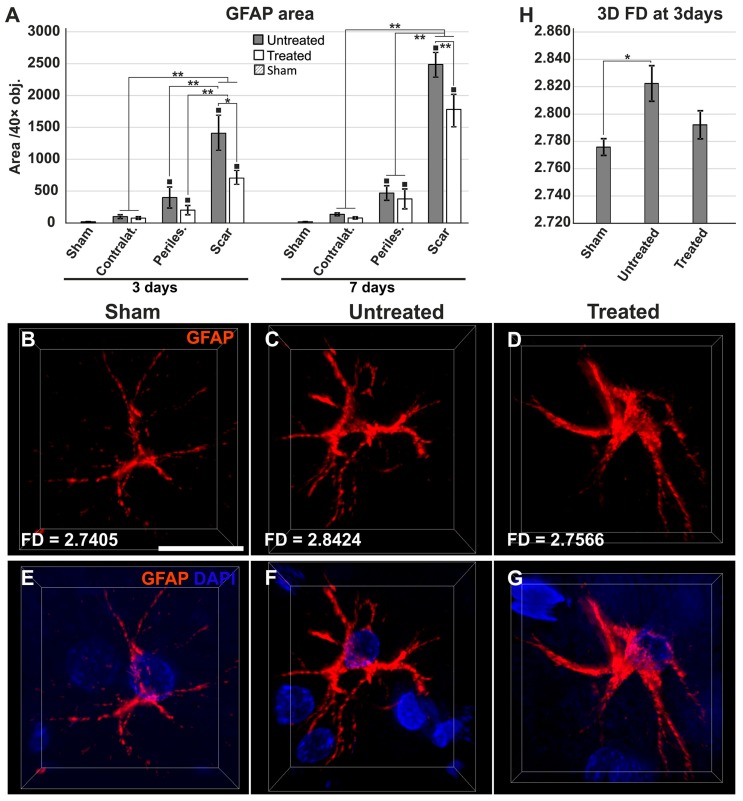
Increased astrogliosis and different astroglial morphologies in untreated animals compared to the treatment group. (**A**) Gliotic areas increase from the contralateral hemisphere to the ipsilateral hemisphere and the scar region, where they are significantly higher for untreated animals compared to treated rats, for 3-day and 7-day survival times (* *p* < 0.05, ** *p* < 0.01; ^■^ *p* < 0.01 (for pathological regions versus sham animals), using a one-way ANOVA followed by a post hoc Fisher’s LSD test, *n* = 5–6/group). The differences between 3 and 7 days are not included for reasons of clarity; (**B**–**D**) Representative three-dimensional projections of scar astrocytes show different morphologies and 3D fractal dimension values (FD) for the sham, untreated, and treated animals at 3 days; **E**–**G** images include also the DAPI nuclear morphology for better orientation; (**H**) Due to the high diversity of the astrocyte morphologies, three-dimensional FD values could only differentiate between sham and untreated astrocytes (F(2,16) = 3.843, * *p* = 0.047). Data are expressed as the means ± SEM. Scale bars in the micrographs represent 20 µm.

Next, we evaluated GFAP/albumin/AQP4 in control, sham, treated, and untreated animals. Increasing albumin infiltration in the perilesional cortices of treated/untreated animals with 3- and 7-day survival times was associated with increased gliosis and a transition of aquaporin expression from vessels to neuropil. When we quantified the expression areas of GFAP, as expected, the higher scar density was recorded for the animals surviving 7 days, than those that had survived 3 days, and this held true for both treated and untreated groups, although TGN-020 injected animals tended to exhibit lower gliosis compared to uninjected rats (Figure 3A). However, the glial reaction in the scar was wider in untreated animals compared to those injected with TGN-020, and these differences were significant both at 3 days (1427.76 ± 276.87 µm^2^/40 × objective (obj.) versus 738.32 ± 103.18 µm^2^/40 × obj.) (*p* < 0.05) and at 7 days (2487.47 ± 197.997 µm^2^/40 × obj. versus 1782.18 ± 260.132 µm^2^/40 × obj.) (*p* < 0.001) survival times.

On higher magnification of individual astrocytes from within the reactive glial rim, in both treated and untreated animals, these cells exhibited clear activated profiles with more abundant cytoplasm, eccentric nuclei, and blunter and less ramified processes, compared to the rare glial cells that could be identified in the sham and control animals, which exhibited thin, elongated, and less branched extensions (Figure 3B–G). As we could clearly distinguish the processes of each individual astrocytic cell at only 3 days survival time, we performed a comparative 3D fractal analysis in an attempt to quantify any differences in the fine morphology of these cells (Figure 3H). Although the complicated and non-constant morphology of the cells would not allow a complete, clear-cut differentiation among sham, treated, and untreated animals, astrocytes in untreated animals showed significantly higher fractal dimension (FD) values (2.8224 ± 0.0318) compared to the sham astrocytes (2.7758 ± 0.0122), while those of treated rats exhibited intermediate values (2.7921 ± 0.0289) (*p* < 0.005). Treated animals showed, thus, a lower albeit not significantly different FD average value compared to the untreated animals, showing a tendency for less complex architecture in these cells, closer to that of sham astrocytes.

### 2.5. Reduced Oedema in TGN-020-Treated Animals

**Figure 4:**
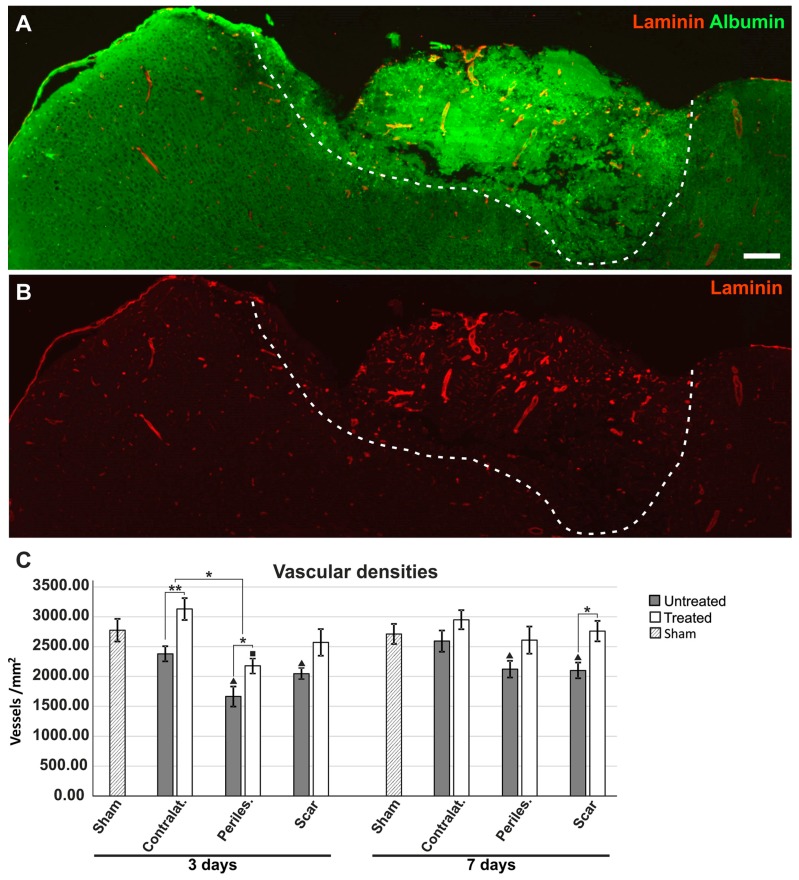
Increased vascular densities in TGN-020-treated animals. (**A**,**B**) Immunohistochemistry for albumin and laminin-1 reveal increased/conserved laminin expression in the infarcted areas (delineated by a dotted line) and adjacent tissue, in a 3-day post-MCAO TGN-020-treated animal; (**C**) Vascular densities show increased values for 3-day treated animals for the contralateral hemisphere and perilesional ipsilateral hemisphere, while in 7-day surviving animals, the difference was significant only for the scar tissue (* *p* < 0.05, ** *p* < 0.01; ^■^ *p* < 0.05 and ^▲^ *p* < 0.01 (for pathological regions versus sham animals), using a one-way ANOVA followed by a post hoc Fisher’s LSD test, *n* = 5–6/group). The differences between 3 and 7 days are not included for reasons of clarity. Data are expressed as the means ± SEM. Scale bars in the micrographs represent 200 µm.

The immunoreactivity for laminin was still intact in all ischemic and perilesional areas, so we utilized this staining to assess the vascular densities as an indirect measure of interstitial oedema (Figure 4A,B). At 3 days after the ischemic event, untreated animals had significantly lower vascular densities in the surrounding ipsilateral hemisphere (1663.51 ± 168.01/mm^2^) and the contralateral cortex (2379.94 ± 126.88/mm^2^), compared to treated animals (2168.94 ± 113.70/mm^2^; 3130.73 ± 182.38 mm^2^) (*p* < 0.05), with the TGN-020-treated animals showing a tendency for higher average values, although not significantly different from those of the sham group (Figure 4C). Treated animals showed even higher, but still not significantly different vascular densities compared to sham tissue, for the contralateral cortices. At 7 days, for untreated animals, vascular densities increased slowly for the ipsilateral and the contralateral hemisphere areas, but for the gliotic scar (2100.85 ± 132.74/mm^2^), the values were still significantly lower than those of the treated animals (2760.24 ± 172.04/mm^2^) (*p* < 0.05). For TGN-020-treated animals, all vascular densities became more uniform toward the average values for the sham tissues.

### 2.6. Effects of AQP4 Inhibition on the Pathways for Intramural Periarterial Drainage

**Figure 5:**
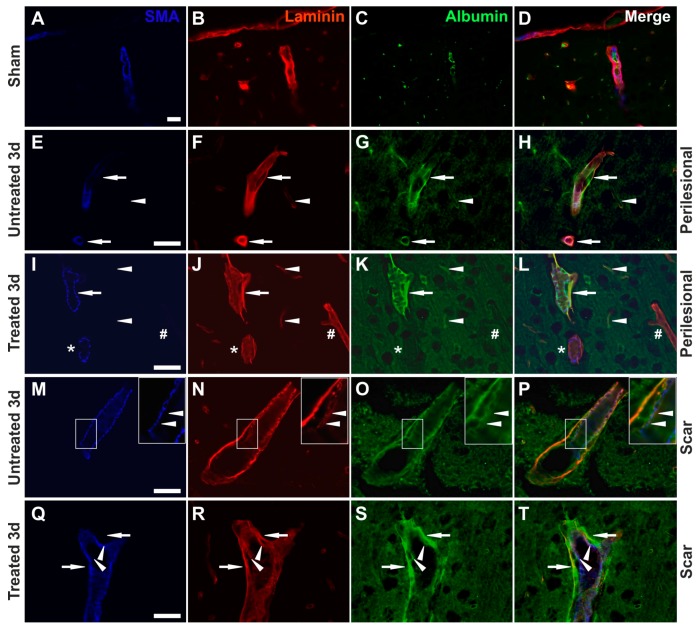
Increased retention of albumin along the vascular basement membranes. (**A**–**D**) An exemplary image from a sham animal shows endogenous rat albumin being present only in the vascular lumen for an arteriole (SMA-positive) and surrounding capillaries; (**E**–**H**) In untreated MCAO animals, arterioles (arrows) and capillaries (arrowhead) show albumin colocalizing with laminin on their basement membranes, and frequently these basement membranes with albumin deposits are thickened (arrows); (**I**–**L**) In TGN-020-treated MCAO animals, arterioles (arrow) and capillaries (arrowheads) also show albumin colocalizing with laminin in their external basement membranes, and these basement membranes with albumin deposits are even thicker here (arrow). Not all SMA-positive (*)/SMA-negative (#) vessels have albumin deposits; (**M**–**P**) Albumin colocalization occurs in arterioles in the exterior basement membranes, and less in those of the tunica media and under the endothelium (arrowheads), (**Q**–**T**), but to a lesser extent than in the external basement membranes for the treated animals (arrowheads versus arrows). Scale bars in the micrographs represent 40 µm.

**Figure 6:**
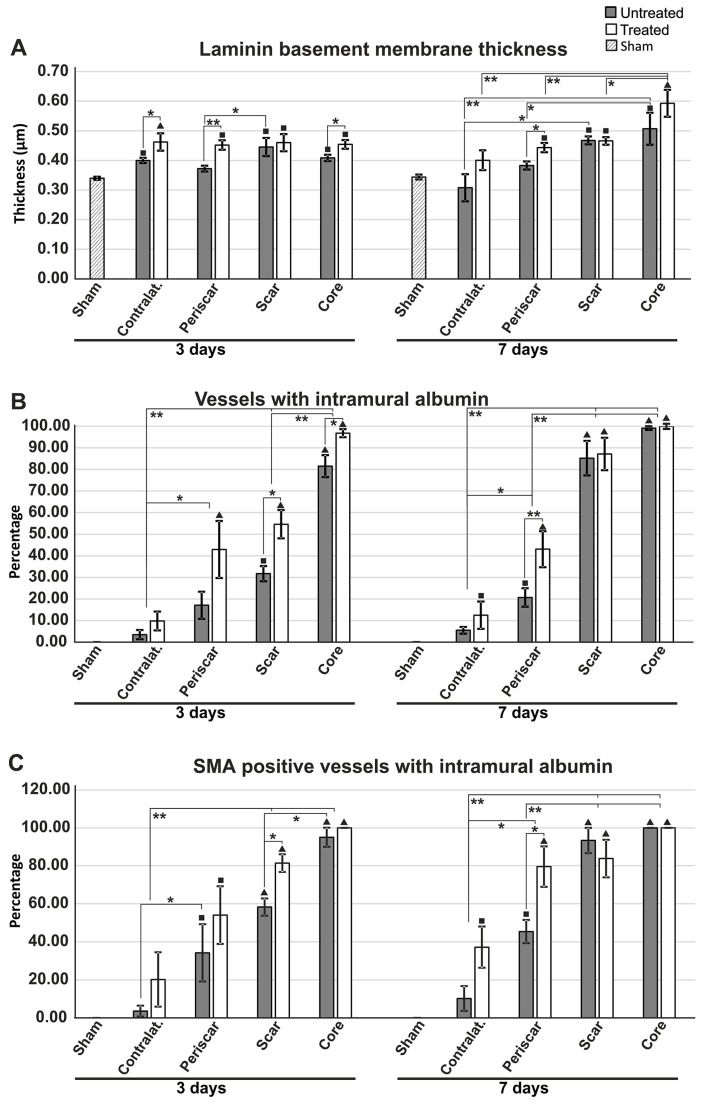
Characterization of the vessels with intramural albumin retention. (**A**) Direct measurement of the outermost basement membrane based on laminin immunohistochemistry reveals an increased thickness for treated animals compared to untreated animals, especially at 3 days after MCAO (in the infarct core, the ipsilateral hemisphere around the gliotic scar, and the contralateral hemisphere), while this difference was significant only for the ipsilateral peri-scar tissue at 7 days of survival; (**B**) There were more albumin-positive vessels in treated animals than in untreated ones for the infarct core and scar region at 3 days after MCAO, and in the peri-scar regions at 7 days after MCAO. Overall, there was an increase in the number of albumin-positive vessels from the distant contralateral hemisphere to the infarct core; (**C**) The percentage of larger muscular vessels (SMA-positive) being stained for albumin increases from the contralateral hemisphere to the infarct core, with a tendency toward higher values in treated animals (* *p* < 0.05, ** *p* < 0.01; ^■^ *p* < 0.05 and ^▲^ *p* < 0.01 (for pathological regions versus sham animals), using a one-way ANOVA followed by a post hoc Fisher’s LSD test, *n* = 5–6/group). The differences between 3 and 7 days are not included for reasons of clarity. Data are expressed as the means ± SEM.

The basement membranes of the endothelia were distinct from the outer parenchymal basement membrane and more intensely stained in infarcted areas compared to contralateral hemispheres and sham animals (Figure 4A,B and Figure 5A–T). Moreover, while in sham and control animals, laminin expression was restricted to thin, well-defined, fused basement membranes for both capillaries and arterioles (Figure 6A–D), in perilesional ipsilateral cortices and scar regions, these basement membranes appeared thicker, and with two clearly distinguishable layers for arterioles (Figure 5E–T).

Moreover, while endogenous albumin was kept inside the lumen of the blood vessels in control animals, there were many vessels showing a clear-cut colocalization of albumin and laminin in the MCAO animals for both the untreated and treated lots, and this albumin entrapment appeared to become more intense in the perilesional cortex of the scar region (Figure 5A–T). In larger vessels, with enlarged perivascular spaces, albumin entrapment was present at the level of the glial-pial basement membranes, but did not colocalize with smooth muscle actin.

We next aimed to assess the thickness of the basement membranes by directly measuring the laminin-1 staining only on the glial–pial basement membrane layer independently visible in fluorescence microscopy. Although there was a general tendency for all MCAO animals to have thicker basement membranes compared to sham animals, this increase was even more pronounced in TGN-020-treated animals compared to non-treated animals (Figure 6A). At 3 days survival time, the treated animals had significantly thicker basement membranes compared to untreated animals (F(8,42) = 4.128, *p* = 0.002) in the contralateral hemisphere (0.462 ± 0.029 μm versus 0.400 ± 0.009 μm, *p* < 0.05), the ipsilateral peri-scar tissue (0.452 ± 0.016 μm versus 0.373 ± 0.010 μm; *p* < 0.001), and the necrotic core (0.450 ± 0.010 μm versus 0.410 ± 0.010 μm; *p* < 0.05). At 7 days survival time, treated animals (0.443 ± 0.016 μm) had significantly thicker basement membranes compared to untreated animals (0.383 ± 0.014 μm), only for the peri-scar tissue (*p* < 0.05).

We next evaluated the total number of vessels with intramural albumin staining, regardless of their diameter (Figure 6B).

The percentage increased clearly from the contralateral hemisphere to the infarct core, where almost all of the blood vessels were also immunopositive for albumin in their walls. Interestingly, in almost all instances, there was a tendency for the treated animals to have more albumin-positive vessels, with significant differences for the core (96.818 ± 1.841% versus 81.556 ± 5.120%; *p* < 0.05) and scar tissue (54.635 ± 6.544% versus 31.715 ± 3.563%; *p* < 0.05) at 3 days, and only for the peri-scar tissue (43.073 ± 8.351% versus 20.728 ± 4.288%; *p* < 0.001) at 7 days of survival.

We also sought to assess any putative differences in albumin intramural positivity for the larger SMA-positive vessels (Figure 6C). Again, the trend was an increase from the contralateral hemisphere to the infarct core, with a tendency for the treated animals to have larger percentages. Significant differences could be recorded in the glial scar areas at 3 days (81.333 ± 4.653% versus 58.254 ± 4.531%; post hoc Fisher’s LSD test, *p* < 0.05) and in the ipsilateral hemisphere peri-scar tissue at 7 days of survival (79.524 ± 10.796% versus 45.370 ± 6.079%; *p* < 0.05).

### 2.7. Reduced Apoptosis in TGN-020-Treated Animals

**Figure 7:**
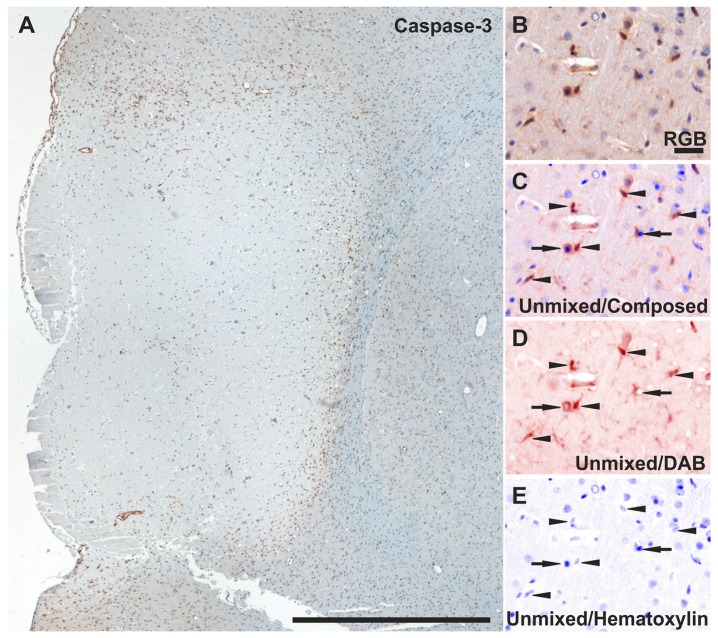

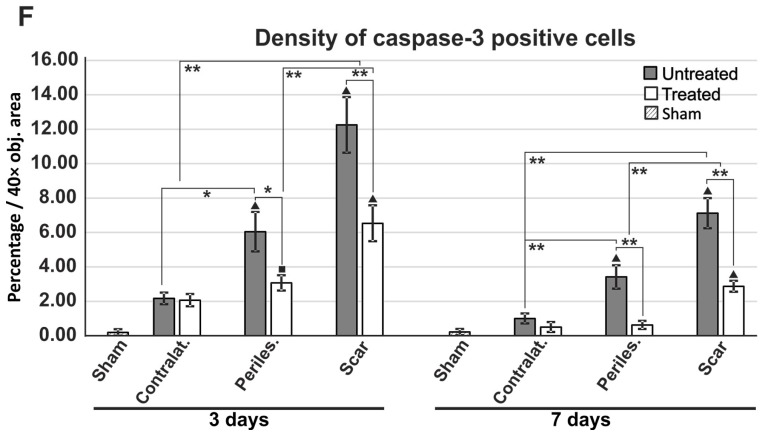
Assessment of apoptosis by counting cleaved caspase-3-positive cells. (**A**) Low-magnification overview of cleaved caspase-3 expression pattern in a 3-day untreated MCAO animal. (**B**) Representative perilesional higher-magnification RGB images decomposed as pure DAB and hematoxylin signals after spectral unmixing (**C**–**E**) showing that only cells presenting immunoreactivity in the nucleus (arrowheads) were counted for the analysis, while cells immunopositive in the cytoplasm only were rejected (arrows); (**F**) Direct counting revealed lower apoptosis levels for treated animals in the ipsilateral perilesional hemisphere and the scar areas at 3 days and 7 days after injury (* *p* < 0.05, ** *p* < 0.01; ^■^ *p* < 0.05 and ^▲^ *p* < 0.01 (for pathological regions versus sham animals), using a one-way ANOVA followed by a post hoc Fisher’s LSD test, *n* = 5–6/group). The differences between 3 and 7 days are not included for reasons of clarity. Data are expressed as the means ± SEM. Scale bars in the micrographs represent 1 mm (**A**), and 20 µm (**B**–**E**).

Lastly, to evaluate whether the AQP4 blocker might have any protective effect after MCAO, we directly counted all cleaved caspase-3 nuclear-labelled non-luminal endothelial-like cells in centripetal regions, starting from the glial scar (Figure 7A–E).

At 3 days survival time, there was a markedly lower number of cleaved caspase-3-positive cells in the TGN-020-treated animals for both perilesional (3.06 ± 0.44%) and glial scar (6.533 ± 1.044%) regions compared to untreated animals (6.04 ± 1.14%; *p* < 0.05, and 12.25 ± 1.61%, *p* < 0.001) (Figure 7F). This difference was also conserved in the same two regions between the treated (0.62 ± 0.23%; 2.87 ± 0.31%) and untreated 7-day survival animals (3.41 ± 0.68%, *p* < 0.001; and 7.12 ± 0.87%, *p* < 0.001), even if the total number of apoptotic cells decreased in all regions between 3 and 7 days for both animal groups.

## 3. Discussion

While specific and independent AQP4 modulators are still elusive, a relatively recent screening study identified *N*-(1,3,4-thiadiazol-2-yl)pyridine-3-carboxamide dihydrochloride (TGN-020) as a potent AQP4 and AQP1 inhibitor [18]. Considering that AQP4 is distributed in humans, and especially in rodents, to the subpial and perivascular end-feet of astrocytes [12,21], and that AQP1 is expressed mainly in the choroid plexuses epithelium [22], the effects of TGN-020 on fluid intake buffering in the brain must be mainly a consequence of AQP4 modulation [23]. In a mouse model of focal cerebral ischemia, a single pretreatment dose with TGN-020 at 15 min before the induction of ischemia significantly reduced brain swelling without any detectable side effects [19]. However, there is no histopathological assessment for effects of TGN-020 administered after the onset of ischemia, as would be necessary in order for it to be considered a potential treatment target for ischemic stroke in humans.

TGN-020 inhibits AQP4 in vitro with a half maximal inhibitory concentration (IC_50_) of 3 μM [18]. In animal and human trials utilizing the 11C radiolabeled ligand ([11C]TGN-020) for visualizing AQP4 in Positron Emission Tomography (PET) imaging, the authors have shown that a 1 μg/kg (~4.5 pM/kg) dosage exhibits a first-pass effect peak within the first 10 min after intravenous injection, followed by a lower plateau phase for absorption in the cortex and choroid plexus in the interval of 15–60 min [20,24]. The fact that larger dosages of 1, 10, 100, and 200 mg/kg still did not show any clear toxic effects in different studies, including this one, ensured that our dosage at 15 min after MCAO (100 mg/kg) would fall into an effective dosage range, and would still cover the first few hours after ischemia induction, when the entire cytotoxic oedema phase occurs [25].

Here, we have first sought any evident histopathological changes and modifications in the expression patterns of AQP4 in organs known to express it, such as the brain, kidneys, eyes, and gut (see Supplementary Results, Figures S2–S3). Unchanged general histology and astrocyte culture cellular morphology were also reported by other studies utilizing TGN-020 or small interfering RNA targeting AQP4 (siAQP4) [20,26]. At 3 and 7 days after a single intraperitoneal administration of TGN-020, there was no change in the histology of these organs, nor in the expression patterns of AQP4 in the absence of MCAO.

On the other hand, hemorrhagic transformation is more common in reperfusion models and human large ischemic strokes, where high pressure blood reflow permeates through damaged BBB, and in these conditions, AQP4 inhibition would aggravate the vasogenic oedema [27]. This, together with the fact that the infarct volume and blood–brain barrier disruption are accentuated by permanent MCA occlusion compared to 1–2 h temporary occlusion [28], oriented us to choose a permanent MCAO model to explore the full potential of AQP4 inhibition of non (or minimal)-vasogenic oedema.

In our experimental setup, after the ischemic event, in the perilesional areas, AQP4 expression became more diffuse in the neuropil at 3 days without losing its perivascular localization, while at 7 days, it increased mainly in the neuropil of the scar, but still being present within the perivascular compartment further away from the infarct. We and others have previously shown that after an ischemic event, AQP4 expression shifts from the perivascular sector toward the full astrocytic membrane, as demonstrated by high colocalization rates with another astroglial membrane marker, glutamate transporter-1 (GLT-1) [12,29], and now it seems that TGN-020 amplifies this glial shift from the perivascular end-feet at the level of the glial scar tissue.

The integrity of the blood–brain barrier (BBB) is compromised following stroke [30], leading to the exposure of the CNS to blood-derived factors that are not normally present in the neuropil. Since TGN-020 absorption in the CNS has been showed to exhibit a peak within the first 10 min after an intravenous injection [20], and there was no noticeable increase in albumin capture by cellular compartments, except blood vessel walls outside the core and the scar area at 7 days, we aimed to characterize, more closely, albumin leakage into the parenchyma in TGN-020-treated animals versus untreated counterparts at 3 days after MCAO. A distance frequency analysis distribution revealed that in treated animals, albumin-positive cells were less frequent and more homogenously distributed at different distances from the infarct core, while for untreated animals, there was a spike in the distribution of these cells between 500 and 1000 µm. To our knowledge, this is the first direct histopathological assessment reduced fluid leakage into the brain of TGN-020-treated animals [19]. Not only does albumin uptake decrease with time after ischemia with no hemorrhagic transformation, but other studies have also shown a relatively rapid albumin degradation at 5 days after uptake [31]. We next evaluated the extent to which the TGN-020 treatment might influence the density of the glial scar around the infarct core by a direct measurement of the GFAP-stained areas. As expected, the glial scar increased in density from 3 to 7 days, but for treated animals, there was a significant reduction in density at both time points. Such a reduction in gliosis might facilitate tissue regeneration following injury, and it has been shown that decreasing the glial scar improves neural progenitor cell migration toward the lesion [32,33]. However, astrocytes are also the main cells that uptake water and glutamate [26,34], so we hypothesized that discrete swelling and more robust architecture of their processes might be identified in our treated animals. For this reason, we isolated individual astrocytes on a high-magnification objective, and calculated the three-dimensional fractal dimension (3D FD) of individual cells from the scar region at 3 days’ survival time (see Supplementary Materials and Methods). Although the complex and stochastic morphology of the astrocytes did not allow clear-cut differentiation between treated and untreated animals, the average 3D FD values were significantly larger in untreated animals compared to sham animals, with the treated animals exhibiting intermediate values. This might be translated into a less complex architecture of the glial arborization in treated versus untreated animals, suggesting that blunter, thicker, and less ramified processes might result after water uptake into the glial cells in TGN-020-treated animals. By directly counting cleaved caspase-3-expressing cells with a non-vascular phenotype, we have shown next a clear-cut reduction in the number of apoptotic cells in the scar and peri-scar regions of the treated animals, even at 7 days after induction of the MCAO, and this non-transient reduction of apoptosis also translated into an improvement in later motor outcome after ischemia. We have recently showed by direct ex vivo three-dimensional scanning of fresh brains, that TGN-020 administrated at 15 min after MCAO decreases infarct volumes and total telencephalon volumes compared to untreated animals [35]. AQP4 inhibition has been also been shown to increase cerebral blood flow, and these mechanisms clearly add up to the resulting neuroprotective role demonstrated here [36].

Utilizing vascular density as a surrogate for assessing interstitial oedema, we have shown here that immediate post-MCAO administration of TGN-020 increased vascular densities in all studied regions in the acute phase of the lesion (i.e., at 3 days), and with a significant increase still being detected in the scar region at 7 days of survival.

**Figure 8:**
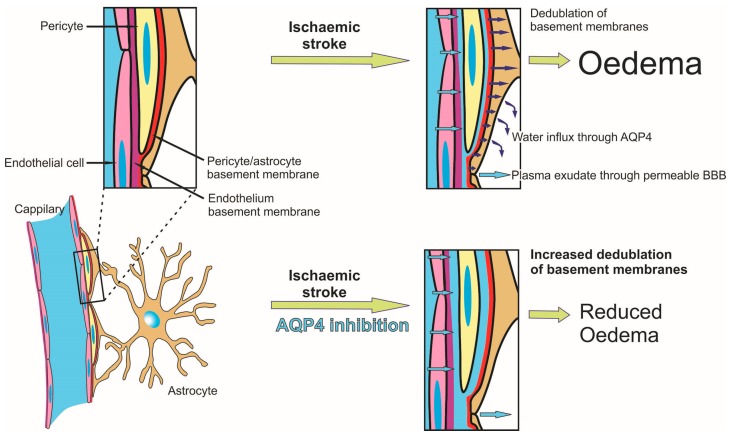
Overview of the mechanism driving reduced oedema and stasis in the paravascular interstitial drainage pathway following aquaporin-4 inhibition in ischemic tissue. Following ischemia, hypoxic endothelial cells lose their tight junctions and increase their permeability for serum, which infiltrates and splits vascular basement membranes, and finally infiltrates through/between the astrocytes into the parenchyma. As the mechanism of water transport at the level of the pericyte/astrocyte basement membrane is mainly dependent on AQP4, this influx is significantly reduced after its inhibition, leading to reduced water infiltration into the parenchyma, and consecutive further splitting of the basement membranes.

Vascular basement membranes are essential and complex structures that support all the elements of the BBB, and it has been shown that the expression levels of laminin are transiently upregulated after MCAO [37,38]. Our immunohistochemistry and direct measurement data revealed that the laminin staining intensity increase is, in fact, due to an increased thickness of the vascular basement membranes, especially for the ischemic core and the peri-scar tissue, in all MCAO animals. Moreover, treated animals tended to show thicker basement membranes for both 3- and 7-day survival, except in the scar region, where the differences might have been alleviated by the retracting glial scar. Further on, most of the vessels in the core region showed intramural albumin staining colocalizing with laminin, or situated within already described duplications and folds of the basement membranes in inflammatory conditions [39]. This intramural albumin staining decreased further away from the infarct core, but was constantly more frequent in the vessels of the TGN-020-treated animals, especially in the acute phase of the stroke. Albumin was present in the glial–pial basement membranes or between the endothelial and glial–pial basement membranes in virtually all small vessels, in the core and scar regions, with the treated animals tending to exhibit more SMA-positive vessels with intramural albumin reactivity. This suggests that extravasated albumin enters the intramural paravascular drainage pathways surrounding the smooth muscle cells, in an attempt to be cleared away [40]. It has been shown that upon the occurrence of cerebral oedema, vascular basement membranes show glio–basal dissociation, duplication, and funneling toward the endothelial cells, induced by the increased vacuolar and vesicular trans-endothelial transport [41]. Our observations showing increased glial–pial basement membrane thickness, increased colocalization of endogenous albumin with this layer, but not with the membranes around smooth muscle cells, and increased participation of arterioles in this intramural albumin trapping suggests that TGN-020 acts upon the formation of intramural paravascular drainage pathways (Figure 8) [42]. This could have profound implications upon the homeostasis of the cerebrospinal and interstitial fluids, and drainage of peptides such as amyloid-β (Aβ) [43]. In AQP4 null /APP/PS1 mice, there is a decline in cognitive performance, together with increased Aβ deposition and cerebral amyloid angiopathy, when compared to APP/PS1mice [44], most likely as a result of a failure to clear soluble Aβ along the intramural peri/paravascular drainage pathways of the blood vessels [45,46].

The main limitation of the present study comes from the small number of animals utilized here, a direct reflection of the price of the TGN-020 itself. We have utilized laminin staining to assess vascular density, and thus to extrapolate the intervascular distances as an indirect measure of total oedema, even in areas where newly formed vessels might not yet express laminin (the infarct and the scar areas). However, since AQP4 non-permanent inhibition has not been described as interfering with angiogenesis, we judged that this underestimation of the vascular densities would be of the same scale in treated and untreated animals, rendering the data still comparable. Although we could not rule out the inclusion of pro-angiogenic non-luminal endothelial cells when counting caspase-3 positive cells, the facts that VEGF and caspase-3 expression vary inversely during ischemia, together with the fact that AQP1 knockout seems to even reduce angiogenesis, support our methodology [47,48].

To our knowledge, this is the first reported study to have administered a single AQP4 inhibitor after an ischemic event, on a non-AQP4-knockout genetic background, without a direct intracerebral injection, and with detailed histopathology [16,19,49].

## 4. Materials and Methods

### 4.1. Animals

The study was approved by the local ethics committee of the University of Medicine and Pharmacy of Craiova, Romania (agreement 151/24 November 2015), and was performed on 46 male Sprague Dawley rats aged 3–4 months (mean weight 419.35 g, SD = 46.45 g). The animals were housed in groups of three in a controlled 12 h light/12 h dark schedule, and had ad libitum access to water and a standard pellet diet, until the day before permanent MCAO surgery.

To follow the effects of a single TGN-020 dose administered after permanent MCAO, we randomly assigned each animal to one of the following four groups: (i) a treated stroke group (*n* = 6 for 3-day survival, *n* = 6 for 7-day survival); (ii) an untreated stroke group (*n* = 6 for 3-day survival, *n* = 6 for 7-day survival); (iii) a sham control group (*n* = 6 for 3-day survival, *n* = 6 for 7-day survival); and (iv) a healthy control group (*n* = 4). Six more rats were utilized to test for any histopathological changes induced by TGN-020 alone, and after the treatment, the animals were maintained without any surgery for 3 (*n* = 3) or 7 (*n* = 3) days before being euthanized.

We choose to follow up the animals at 3 and 7 days post-MCAO in order to (i) be able to observe morphological changes consecutive to the peak of pooled cytotoxic–vasogenic oedema phase (at 2–3 days) [50], and (ii) to evaluate further cellular changes of the acute phase around the beginning of lesion organization through microglia/macrophage activity (peak at 3–7 days) [51] and gliosis (beginning with 1–2 weeks) [52].

From a total of 36 operated animals, one animal died during the first night after the surgery, and pathology revealed large bleedings at the site of intervention (untreated group, 3-day survival).

### 4.2. Surgery and Sham Animals

Focal cerebral ischemia was induced by a simplified protocol aiming only for the permanent occlusion of the middle cerebral artery. The rats were anesthetized using a ketamine/xylasine cocktail (80–100 mg/kg/5–10 mg/kg intraperitoneally), the head fixed in a stereotaxic device, and a small craniotomy was made in the temporal bone, above the MCA bifurcation [53]. The dura was incised, and the distal MCA was exposed. After being lifted up with a tungsten hook attached to micromanipulators, the MCA was cauterized under a surgical microscope, with a Toolcraft ST-50D digital soldering station, and a 0.2 mm tip heated at 340 °C for 5 s [54], without damaging the pia mater or the underlying cortex itself. The artery was coagulated in two points, leading to a complete absence of blood flow under the microscope. Body temperature was maintained at 37 ± 0.5 °C throughout the procedure using a heating pad. After surgery, the animals were placed on a 37 °C warming tray, with food and water being accessible inside the cage.

### 4.3. TGN-020 Preparation and Treatment

As treatment, the animals received a single intra-peritoneal injection of TGN-020 chloride salt (*N*-(1,3,4-thiadiazol-2-yl)pyridine-3-carboxamide dihydrochloride, M = 279.1463) (Ukrorgsyntez Ltd., Kiev, Ukraine), 100 mg/kg, dissolved in 0.4 mL sterile distilled water, and titrated with 1 M NaOH to a pH of 8, at 15 min after MCAO. Untreated MCAO animals received the same volume of isotonic saline. Sham operated animals were only anesthetized and the skulls drilled to expose the MCA.

## 5. Conclusions

Altogether, the present study clearly demonstrates the benefits of a singular AQP4 inhibition in the treatment of ischemic stroke in a (for now) narrow therapeutic window. An essential result is also that inhibition of AQP4 blocks fluid influx along the glial–pial basement membranes, and demonstrates its important role in the formation of paravascular drainage pathways. Further experiments aiming for longer therapeutic windows after an ischemic event are needed to ascertain AQP4 inhibition as a therapeutic option, as well as studies that would directly assess its influence on the interstitial fluid formation and brain solutes drainage.

## Supplementary Materials

Supplementary materials can be found at www.mdpi.com/1422-0067/19/1/46/s1.

## Abbreviations

Aβ	Amyloid beta peptide
AQP4	Aquaporin-4
BBB	Blood-brain barrier
IOD	Integrated optical density
MCA	Linear dichroism
MCAO	Medial cerebral artery occlusion
siRNA	Small interfering RNA
SMA	Smooth Muscle Actin
TGN-020	2-(Nicotinamide)-1,3,4-thiadiazole AQP4 inhibitor
tPA	Tissue plasminogen activator
