# Supplementary material for: Inhibition of Aquaporin-4 Improves the Outcome of Ischaemic Stroke and Modulates Brain Paravascular Drainage Pathways
Source: Int J Mol Sci. 2017 Dec 23;19(1):46. doi: 10.3390/ijms19010046 (PMC5795996; doi:10.3390/ijms19010046)
Supplement: Supplementary file 1 [file ijms-19-00046-s001.pdf]

Supplementary materials:

1. Materials and Methods

1.1. Rotarod Testing

Motor signs of stroke were evaluated based on a classical rotarod setup. Basically, after being trained for 7 days before MCAO, the animals underwent surgery (day 0) and were re-tested at 3 and 7 days. The rats were allowed to cross freely, a 10 cm diameter 130 cm long rod rotating at 3 and 6 rpm towards a cage, with other animals situated at the other end of the rod. The animals were brought with their cages in the rotarod testing room 30 minutes prior to any testing. Scores indicate the following: 0, rat falls immediately; 1, rat does not move; 2, rat crosses but falls before reaching the target; 3, rat crosses the rod, but the limbs are moved asymmetrically; 4, rat crosses the rod, but the left hind limb is used less than 50% of the necessary time; 5, rat crosses the rod with some difficulties; 6, rat crosses the rod without mistake, with symmetrical movements. Each testing was repeated twice, and the results were averaged and expressed as average ± standard error of the means (SE).

1.2. Tissue Processing and Histopathology

After euthanization, the animals were perfused with 10% neutral buffered formalin, and the brain, eyes, liver, lungs, small intestines, and kidneys were harvested and further fixed for 2 days at room temperature. All organs have been sectioned to allow a better fixation, and the brain was cut frontally at bregma levels +2 mm, and −3mm [1]. All tissues were then processed for paraffin embedding and then cut as 5μm thick serial sections on a rotary microtome HM355S with a waterfall-based section-transfer system (Thermo Scientific Inc., Walldorf, Germany). For the brain, the tissue was cut as a series of 10 × 5 μm thick sections followed by 10 × 20μm thick sections. Thin sections from all organs were first prepared and analyzed following routine hematoxylin and eosin staining (together with cresyl violet for the brain).

1.3. Immunohistochemistry

Single enzymatic immunohistochemistry was first performed for all the antibodies enumerated in Table 1 for the brains of all studied animals, and in addition, the anti-AQP4 antibodies were utilized for staining of all the organs harvested from these animals. Briefly, after antigen retrieval, sections were first incubated for 30 minutes in a 1% hydrogen peroxide solution. The sections were next blocked for 1 h in 3% skimmed milk (Bio-Rad, München, Germany), then they were incubated with the primary antibodies for 18 h at 4°C, and the next day, the signal was amplified for 30 minutes utilizing a species-specific rat-adsorbed peroxidase polymer-based system (Nikirei-Histofine, Tokyo, Japan). The signal was detected with 3,3′-diaminobenzidine (Dako, Glostrup, Denmark), and the slides were coverslipped in DPX (Sigma-Aldrich, Hamburg, Germany) after a hematoxylin staining. Each experiment included control brain tissue as positive controls for the existence of known structures (ie, astrocytes, neuronal silhouettes, and blood vessels, respectively), whereas negative controls were obtained by omitting the primary antibodies.

After confirmation on single immunohistochemistry, triple fluorescent immunodetections were performed in order to study the relationship between albumin extravasation, AQP4 positive astrocytes, laminin, and smooth muscle actin within the vascular walls. To assess the relationship of albumin to AQP4 and astrocyte cytoskeleton (GFAP), two series of slides (4 and 20 μm thick) from all the brains of all animals were processed for antigen retrieval, blocked in 3% skimmed milk (Bio-Rad), incubated with goat-anti-albumin antibody (Abcam) for 18 h at 4°C, amplified for 30 minutes with an anti-goat biotinylated secondary antibody (1:100, Dako), and then detected for further 30 minutes with streptavidin Alexa 488 (1:300, Invitrogen, Life Technologies GmbH, Darmstadt, Germany). After thorough washing in PBS, and one more blocking step in 3% skimmed milk, the slides were incubated

with a mixture of rabbit-anti-AQP4 (Santa Cruz) and mouse anti-GFAP (Antibodies online) for 18 h at 4°C, then visualized with a mixture of goat anti-rabbit Alexa 596 and goat anti-mouse Alexa 555 (1:300, Invitrogen) for 1 hour at room temperature. For the albumin/SMA/laminin immunostaining, the two series of slides were first processed for citrate buffer boiling, then digested with proteinase K for 15 minutes at 37°C (Dako). Unspecific binding sites were blocked with 3% skimmed milk (Bio-Rad), then the goat-anti-albumin (Abcam) primary antibody was added for 18 h at 4°C. The next day, the signal was amplified for 30 minutes with the anti-goat biotinylated secondary (1:100, Dako), and detected for another 30 minutes with streptavidin Alexa 488 (1:300, Invitrogen). After a second blocking step in 3% skimmed milk, a mixture of rabbit-anti-laminin (Abcam) and mouse anti-SMA (Dako) antibodies were added on the slides for 18 h at 4°C, and visualized with a mixture of goat anti-rabbit Alexa 596 and goat anti-mouse Alexa 555 (1:300, Invitrogen) after 1 hour incubation at room temperature. In all cases, the slides were coverslipped with the Vectashield fluorescence anti-fading mounting medium containing DAPI (H-1200, Vector laboratories), and protected with nail polish.

1.4. Image Analysis

Conventional light microscopy and fluorescent images were grabbed utilizing a Nikon Eclipse 90i motorized microscope (Nikon Instruments Europe BV, Amsterdam, The Netherlands) equipped with a Prior OptiScan ES111 motorized stage (Prior Scientific, Cambridge, UK), a 5 megapixel Nikon DS-Fi1 CCD cooled color camera (Nikon), and a high quantum efficiency (pixel size 12.9μm x 12.9μm) monochrome Rolera-XR cooled CCD camera (QImaging, Surrey, British Columbia, Canada), driven by the Image-Pro Plus AMS 7 image analysis software (Media Cybernetics, Bethesda, MD, USA).

Fluorescence pictures for image analysis have been collected randomly from the areas of interest (infarct core, surrounding glial scar, the ipsilateral perilesional area on the outer sides of the scar, and the contralateral hemisphere), based on GFAP immunostaining, which made it easy to identify the core of the infarct surrounded by a collar of reactive astrocytes. Composite images were obtained by sequential scanning of each channel with specific pairs of highly selective filter sets, in order to eliminate the cross-talk of the fluorophores, and to ensure a reliable quantification for DAPI, Alexa 488, Alexa 555, and Alexa 594 spectra (Chroma Technology Corp., Bellows Falls, VT, USA). All images were originally stored in Image-Pro Plus's proprietary format, then they were subjected to a five iterations of blind deconvolution algorithm based on a multi-pass adaptive point spread function subtraction of diffracted light (AutoDeblur, Image-Pro Plus; Media Cybernetics).

For evaluating endogenous albumin diffusion around the infarct areas at 3 days after MCAO, whole lesional hemispheres were scanned on a 10× objective for albumin and GFAP signals, on one slide from each animal (bregma level −1mm), and the necrotic areas were demarcated utilizing a manual segmentation tool in Image ProPlus, using as guidance, the stronger diffuse albumin staining in the infarcted tissue, together with the limiting incipient glial scar. Next, we measured the distance from the designated infarct areas to each of the albumin positive cellular silhouette that could be identified on a distance of 2500 μm from the infarct border, (the average cortical thickness at this level), both above and beneath the core. Counting did not differentiate between neuronal and glial cells, and only the cortex area has been considered (thus, no cell was considered beneath the corpus callosum into the striatum or thalamic nuclei), and also no albumin positive vessels were included in this count. The number of albumin positive cells was then plotted as frequency of occurrence against the distance intervals in which they occurred, as a distance-frequency graph [2], and the cellular density was normalized for 1 mm^2.

For vascular densities, blood vessels stained for laminin (being part of the albumin–laminin–SMA series of immunostainings described above), were counted on six 60× aleatory images (area of 18742.52 $μm^2$) (oil immersion, Nikon plan apochromat VC, NA = 1.40) taken in the respective areas of interest, normalized for 1 mm^2, averaged, and reported for the respective region/animal. Moreover, we measured for each vessel, the maximum diameter and thickness of the basement membranes based on the laminin staining, and we have also evaluated its relationship with the endogenous albumin immunostaining, deeming the albumin localization as being present only in the lumen of the vessel

(intravascular), in the wall of the vessel (intramural), and/or in the perivascular space and neuropil. For a correct evaluation of the basement membranes separating the vessels from the neuropil, in thicker vessels with discernable perivascular spaces, we have considered only the external layer component of the basement membrane. In order to assess, correctly, the basement membrane thickness in small arterioles and capillaries, no tangential sections have been considered (i.e. only vessels with clear lumina were considered). Exemplary areas were scanned as z-stacks in the thickness of the specimen, deconvoluted, and visualized utilizing the 3D Constructor module in Image ProPlus.

In order to assess the area of gliosis, in the same series of deconvoluted images, GFAP signal was thresholded based on the Trainable Weka Segmentation plugin [3] in the Fiji image analysis package [4], and the areas were finally measured in Image ProPlus, and reported for the 40× objective area (42191.19 μm^2) (oil immersion, Nikon plan apochromat, NA = 0.95).

For evaluating the tortuosity of glial cells processes surrounding the infarct core at 3 days after the ischemic event, aleatory chosen individual astrocytes from 20 μm thick GFAP stained slides were imaged as complete z-stacks with a z-step of 0.5 μm under an apochromatic 100× objective (oil immersion, Nikon plan apochromat, NA = 1.4). The stacks were further deconvoluted utilizing a five iteration blind deconvolution algorithm, and then each stack was analyzed section by section, manually removing all GFAP signals that did not show continuity with the single chosen astrocyte, ensuring thus that a single cell would be analyzed from this point onward. The remaining signals were automatically thresholded utilizing the Trainable Weka Segmentation plugin in order to ensure the same cut-off parameters, and the resulting series of images were finally binarized and fed to a three dimensional fractal analysis software designed to evaluate the complexity of a three dimensional object by looking at the stacked sectional planes (Fractal analysis system version 3.4.7, developed by Hiroyuki Sasaki, PhD, National Agriculture and Food Research Organization (NARO), Japan).

In order to evaluate the AQP4 and cleaved caspase-3 immunoreactivity in the peri-necrotic scar region, peri-scar and contralateral hemisphere, light microscopy images were grabbed on the same microscope with the 40× objective, utilizing a Nuance FX multispectral camera together with the Nuance analysis software (Perkin Elmer, Hopkinton, MA, USA). After building a custom spectral library from individual slides stained with either hematoxylin or for cleaved caspase-3 (with DAB, as described above), we were able to efficiently count only the cells that clearly showed cleaved caspase-3 staining in their nuclei, regardless of the cytoplasmic staining. All cells were counted on the respective objective area utilizing this approach, except endothelial cells, which were not considered here, and reported as averaged values per region and animal group. For assessing the intensity of AQP4, monochrome images representing the spectral signature of the staining were further utilized to calculate the integrated optical density of the signal (IOD, a dimensionless parameter calculated as the area of the object × average signal intensity) in Image ProPlus.

1.5. Data Analysis

All the data were represented graphically and further analyzed utilizing Microsoft Excel 2013 and SPSS 10.0 (SPSS, Inc., Chicago, IL, USA). Data are represented as average ± SE (standard error of the means), unless specified otherwise. A one-way ANOVA (ANOVA—analysis of variance) with the post hoc Fisher's LSD test was utilized for multiple comparisons. In order to compare the density of albumin-positive cells and the average frequencies for the distance-frequency maps between treated and untreated animals, Student's t test has been utilized. In all cases, $P < 0.05$ was used to indicate statistical significance.

2. Results

2.1. Pathological Confirmation of the Infarct Areas

Histopathology analysis confirmed on all MCAO surviving animals, typical neuropathological changes associated to cortical temporal ischemic strokes. At 3 days, the infarcted areas became paler

than the surrounded tissue, the neuronal cytoplasms lost their distinct Nissl body patterns, sometimes taking a distinct eosinophilic appearance, their nuclei became pale, pyknotic or underwent karyorrhexis (Supplementary Figure S1A, a1). A few foamy macrophages could already be identified at the periphery of the infarcted area at this time (Supplementary Figure S1A, a2). At 7 days, clear-cut liquefaction necrosis was present, with the entire area filled with foamy macrophages and cellular debris (Supplementary Figure S1B, b1). At the edge of the infarct, classical gemistocytic astrocytes could be identified in the neuropil (Supplementary Figure S1B, b2).

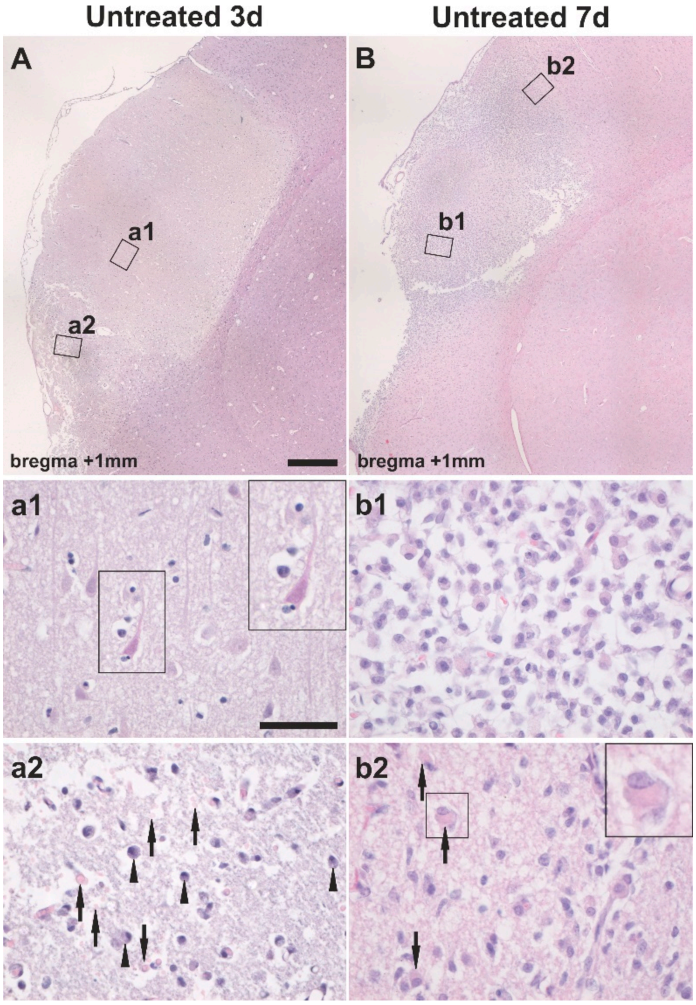

Figure S1. Histopathological confirmation of ischemic stroke without consistent hemorrhagic transformation. (A) At 3 days after MCAO, the infarcted area is paler, visibly demarcated from the rest of the tissue, with (a1) pale neurons, on occasion with a "hypoxic eosinophilic appearance" and loss of Nissl bodies (inset). (a2) At the edge of the infarct, a few foamy macrophages are already present at this time (arrowheads), with minute hemorrhage transformation consisting of not more than a few isolated extravasated red blood cells (arrows). (B) After 6 days, the necrotic area is liquefied, filled with (b1) foamy macrophages, and with (b2) gemistocytic astrocytes at the edge of the infarct (arrows and inset). Hematoxylin and eosin staining, scale bars represent respectively 500 µm (A, B) and 50 µm (a1, a2, b1, b2).

In all these surviving cases, only on occasion could minimal petechial extravasated red blood cells be identified in the core of the lesion or around it, confirming this technique as prone to producing ischemic infarctions without significant hemorrhagic transformation, due to reduced reperfusion after the ischemic event. All infarct areas, at both examination times, were restricted to the cortex, and did not involve structures below the corpus callosum (i.e. striatum, thalamus).

2.2. *General Histopathology and Aquaporin-4 Expression Patterns*

We have next checked all harvested control organs prelevated from animals treated with TGN-020 (without any further surgery) and from untreated control animals, for any histopathological changes or changes in the immunohistochemical staining patterns of aquaporin-4 (Supplementary Figures S2-3). There was no histopathological difference or change between these animals with regards to the histology of their brains, lungs, livers, kidneys, eyes, and intestines. In the kidney, immunohistochemistry showed aquaporin-4 as being expressed in the tubules, and especially in the collective ducts (Supplementary Figures S2A–C), with a membranous-like appearance mostly on the basolateral surface of the collector duct cells.

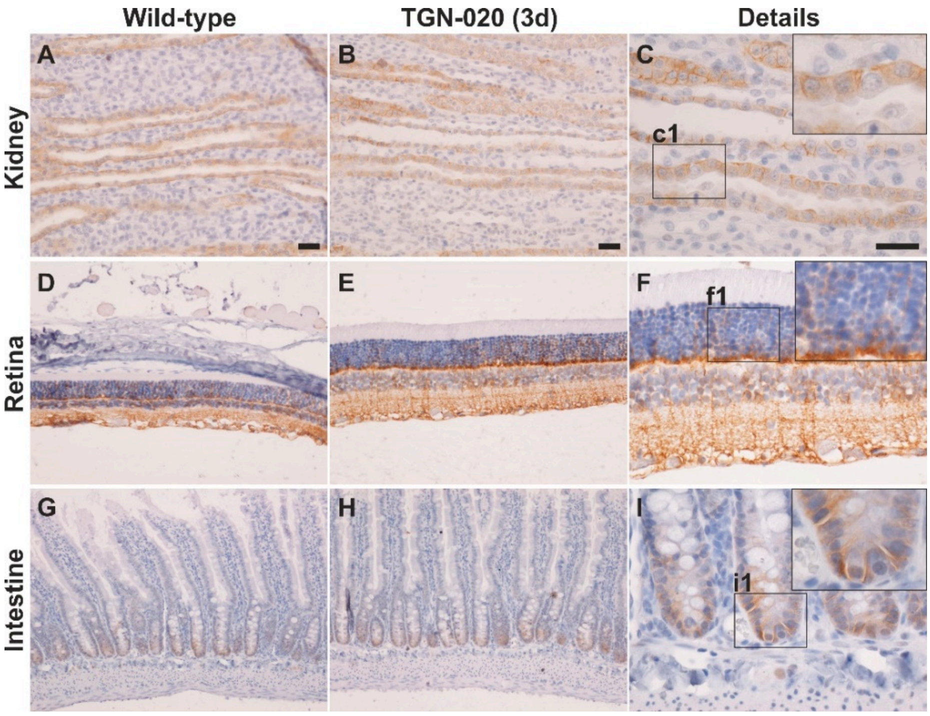

Figure S2. Expression pattern of AQP4 in peripheral organs is not changed by the administration of TGN-020. (A–C) In the kidneys, the water channel is detected on the basolateral surface of the collector duct cells, (D–F) In the retina it appears dense-diffuse in all the internal layers until the perinuclear cytoplasm of the photoreceptor cells, and with no signal in the rod and cones layer. (G-I) In the small intestine, it is present in the basal regions of the Lieberkühn glands, showing membrane-like patterns in the enterocytes. Rectangles represent enlargements of their respective insets. Scale bars represent 20 µm.

In the retina, it was expressed dense-diffusely in all the internal layers until the outer nuclear layer, where only scant signals could be identified in the perinuclear cytoplasm of the photoreceptor cells, and with no signal in the rod and cones layer (Supplementary Figures S2D-F). In the small intestine, the water channel was present especially in the basal regions of the Lieberkühn glands, showing membrane-like patterns in the enterocytes, but sometimes diffusely in their apical cytoplasm (Supplementary Figures S2G-I).

In the brains of treated/non-treated animals, there was a moderate expression in the walls of the blood vessels, and a dense-diffuse staining in the neuropil, with aggregations around blood vessels or brain cells (Supplementary Figures S3 A–F). The staining was also more intense in the upper and lower glia limitans, and petechial in the striatum. Ependymal cells showed a membranous staining pattern, while the choroid plexus epithelial cells were not stained (Supplementary Figures S3 G–I). In the cerebellum, there was a light diffuse staining pattern in the molecular layer, together with a strong membranous staining for Purkinje and granular cells (Supplementary Figures S3J–L). Overall, there was no observable histo-morphological difference between the expression patterns for control animals

injected with TGN-020 and respectively uninjected animals, in all these organs, at both 3 and 7 days following the treatment.

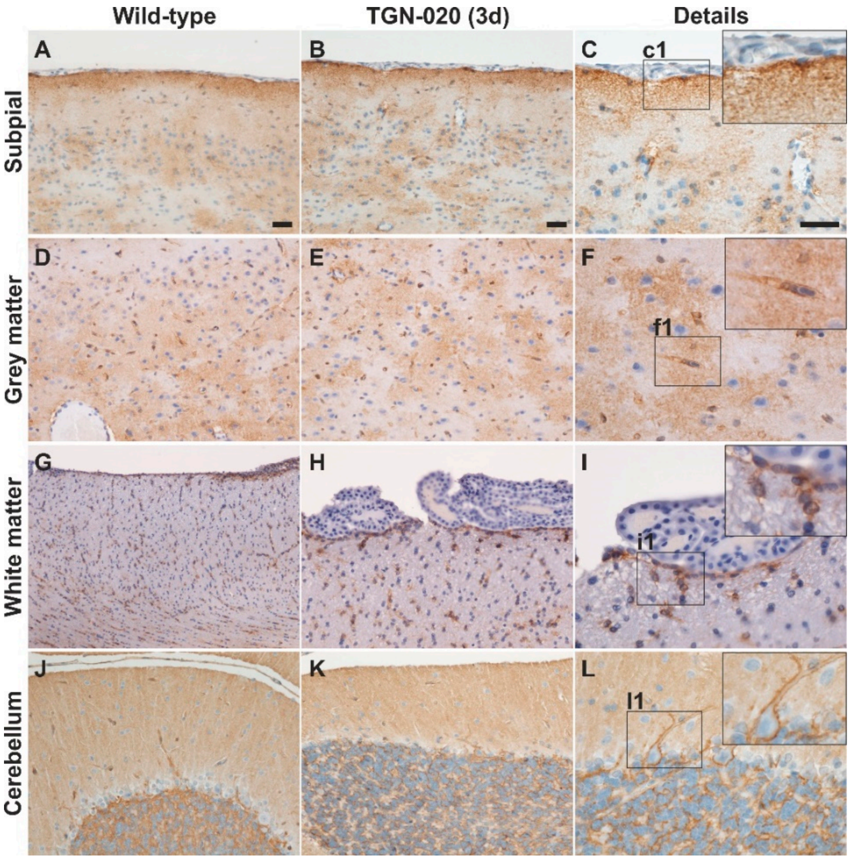

Figure S3. Expression pattern of AQP4 in the brain is not changed by the administration of TGN-020. (A–F) in the gray matter, AQP4 is expressed in the perivascular end-feet, sub-pial external glia limitans, and dense-diffusely on the membranes of the astrocytes. (G–I) In the white matter, its expression pattern is perivascular and in the internal glia limitans, with no reactivity at the level of the choroid plexus, and (J–L) in the cerebellum there was a diffuse staining pattern in the molecular layer associated with a strong membranous staining for Purkinje and granular cells. Rectangles represent enlargements of their respective insets. Scale bars represent 20 μm.

3. References

1. Paxinos, G.; Watson, C., The rat brain in stereotaxic coordinates. 7th ed.; Elsevier: London, 2014.

2. Mogoanta, L.; Ciurea, M.; Pirici, I.; Margaritescu, C.; Simionescu, C.; Ion, D. A.; Pirici, D., Different Dynamics of Aquaporin 4 and Glutamate Transporter-1 Distribution in the Perineuronal and Perivascular Compartments during Ischemic Stroke. Brain Pathol. 2014, 24, (5), 475-493, 10.1111/bpa.12134

3. JS, I. A.-C. A. C. V. K. Trainable Weka Segmentation. http://fiji.sc/Trainable_Weka_Segmentation (10.09.2016),

4. Schindelin, J.; Arganda-Carreras, I.; Frise, E.; Kaynig, V.; Longair, M.; Pietzsch, T.; Preibisch, S.; Rueden, C.; Saalfeld, S.; Schmid, B.; Tinevez, J. Y.; White, D. J.; Hartenstein, V.; Eliceiri, K.; Tomancak, P.; Cardona, A., Fiji: an open-source platform for biological-image analysis. Nature methods 2012, 9, (7), 676-82, 10.1038/nmeth.2019
